# Prior Tooth Mobility and Furcation Involvement Are Associated With Higher Dental Implant Failure Rates: A Propensity‐Matched Cohort Study

**DOI:** 10.1111/clr.70127

**Published:** 2026-04-03

**Authors:** Georgios S. Chatzopoulos, Larry F. Wolff

**Affiliations:** ^1^ Division of Periodontology, School of Dentistry University of Minnesota Minnesota USA; ^2^ Faculty of Dentistry, Health Sciences Aristotle University of Thessaloniki Thessaloniki Greece

**Keywords:** dental implants, furcation involvement, implant failure, mixed‐effects model, periodontitis, propensity score matching, tooth mobility

## Abstract

**Objectives:**

To compare the long‐term survival of dental implants placed in sites with a documented pre‐operative history of severe tooth mobility or advanced furcation involvement against a matched control group.

**Material and Methods:**

This retrospective cohort study analyzed patient records from a multi‐university network. Implants were categorized based on the pre‐extraction site history: Mobility Class (M2), Mobility Class (M3), Furcation Grade (G3), Furcation Grade (G4), and a control group. A 1:4 propensity score matching (PSM) was performed to balance baseline covariates (age, sex, smoking, diabetes, implant arch).

**Results:**

After PSM, a balanced cohort of 3925 implants (785 risk implants, 3140 control implants) was analyzed. The 10‐year survival probability for the matched control group was 98.9%. All risk groups demonstrated significantly lower survival, with M2 at 94.6% and G4 at 95.5%. The adjusted multilevel Cox model confirmed a significantly higher risk of failure for all exposure groups. The adjusted hazard ratio was 2.3 for M2 (95% CI: 1.2–4.4), 3.8 for M3 (95% CI: 1.9–7.5), 3.0 for G3 (95% CI: 1.4–6.5), and 4.5 for G4 (95% CI: 2.1–9.8), compared to the matched controls (all *p* < 0.01).

**Conclusions:**

A pre‐extraction history of severe tooth mobility or advanced furcation involvement at an implant site is significantly associated with long‐term implant failure. Rather than being independent biological causes, these clinical signs likely serve as historical markers for severe localized bone loss and heightened periodontitis susceptibility and should be carefully considered during risk assessment and treatment planning.

## 1 Introduction

Dental implant therapy has become a cornerstone for the rehabilitation of partially and fully edentulous patients, offering a predictable and highly successful long‐term solution for tooth replacement (Pjetursson et al. 2007). However, a significant portion of tooth loss is a direct consequence of severe periodontal disease, making this patient population a common demographic for implant treatment (Serroni et al. 2024). This has led to extensive investigation into whether a history of periodontitis constitutes a significant risk factor for future implant success. The consensus from a substantial body of literature, including numerous systematic reviews and meta‐analyses, confirms that patients with a history of periodontitis are indeed at a higher risk for implant complications, such as peri‐implantitis and implant loss, when compared to periodontally healthy individuals (Annunziata et al. 2025; Carra et al. 2021; Roccuzzo et al. 2010).

The increased susceptibility of periodontitis patients to implant complications is rooted in a shared etiological pathway. Both periodontitis and peri‐implantitis are inflammatory conditions triggered by a dysbiotic oral microbiome in a susceptible host (Smith et al. 2017; Heitz‐Mayfield and Lang 2010). The host's inflammatory response, which leads to the destruction of periodontal tissues around teeth, can be recapitulated around dental implants, resulting in the progressive loss of supporting bone (Schwarz et al. 2018; Renvert and Persson 2009). This inherent predisposition underscores the challenges in achieving long‐term implant stability and health in this patient cohort (Karoussis et al. 2003). Consequently, a history of periodontitis is now widely accepted as a major risk factor for the development of peri‐implantitis (Giok et al. 2024).

In response to this established risk, the management of patients with a history of periodontitis who are candidates for implant therapy emphasizes the critical need for rigorous pre‐operative periodontal treatment and, most importantly, consistent post‐operative supportive care (Lin et al. 2019; Jepsen et al. 2015). Studies have demonstrated that with a well‐structured and regularly attended supportive periodontal therapy (SPT) program, the long‐term survival rates of implants in these patients can be favorable (Lin et al. 2020; Roccuzzo et al. 2012). However, even with diligent maintenance, the risk of biological complications and implant loss remains elevated compared to periodontally healthy individuals, highlighting that while the risk can be managed, it is not entirely eliminated (Monje et al. 2016).

While the patient's overall periodontal history is a known risk indicator, there is a significant gap in the literature regarding the influence of specific, severe, pre‐existing conditions at the tooth site destined for implant placement. Clinical decision‐making often involves extracting teeth with a hopeless prognosis, characterized by conditions such as Class III mobility or Grades III and IV furcation involvement, which are endpoints of severe localized attachment loss (McGuire and Nunn 1996). The focus of much of the existing research has been on the patient as the unit of analysis, categorizing individuals based on a general diagnosis of “history of periodontitis,” often staged and graded according to classifications (Tomasi et al. 2021). Although this approach provides invaluable insight into systemic risk, it often overlooks the specific conditions of the local implant site and whether the extreme severity of the prior localized defect carries a distinct and higher risk.

This lack of specific, site‐based evidence presents a clinical challenge. Therefore, this study was designed to address this critical gap by analyzing a large, retrospective cohort. The primary aim is to compare the long‐term survival of dental implants placed in sites with a documented pre‐operative history of severe mobility or advanced furcation involvement against a propensity score‐matched control group. We hypothesize that even after adjusting for patient‐level confounders, implants placed in these severely compromised sites will exhibit a statistically significant increase in failure rates.

## 2 Material and Methods

### 2.1 Study Design and Population

This study was a retrospective cohort analysis of patient records from dental clinics of universities contributing data to the BigMouth network, including Harvard University, University of Texas Health, The University of California, San Francisco, University of Colorado, Loma Linda University, University of Buffalo, The University of Iowa, The University of Minnesota, and Tufts University. The data encompassed the period between 2011 and 2022. The study protocol was reviewed by the Institutional Review Board of the University of Minnesota, which waived the need for ethical review and approval (#STUDY00016865, 10/10/2022). The protocol was further reviewed and approved by the BigMouth Consortium for Oral Health Research and Informatics clinical review committee. This study was conducted in agreement with the principles of the Helsinki Declaration of 1975, as most recently revised in 2013.

The study population consisted of adult patients who received dental therapy at the participating university clinics between 2011 and 2022. Patient records were initially screened using Current Dental Terminology (CDT) codes for comprehensive or periodic oral evaluations (D0150, D0120, D0180). From this initial cohort, partially or totally edentulous patients who had received at least one endosteal implant were identified using the CDT code D6010 (Surgical placement of implant body: endosteal implant). This study was conducted and reported in accordance with the Strengthening the Reporting of Observational Studies in Epidemiology (STROBE) guidelines for cohort studies.

### 2.2 Definition of Exposure and Control Groups

Following the identification of all implant placements, a detailed assessment of the pre‐operative periodontal status of each implant site was conducted using historical periodontal charting records. Implants were categorized based on the most severe pre‐extraction condition recorded for the specific tooth site:

- Mobility Class 2 Group: Implants placed in sites with a documented history of Class 2 tooth mobility.
- Mobility Class 3 Group: Implants placed in sites with a documented history of Class 3 tooth mobility.
- Furcation Grade 3 Group: Implants placed in sites with a documented history of Grade 3 furcation involvement.
- Furcation Grade 4 Group: Implants placed in sites with a documented history of Grade 4 furcation involvement.
- Control Group: Implants placed in sites with no recorded history of Class 2 or 3 mobility or Grade 3 or 4 furcation involvement.

For an implant to be included in an exposure group, the periodontal record (mobility or furcation) had to pre‐date the surgical implant placement date. Furcation involvement was classified according to Glickman (1972), where Grade 3 represents severe bone loss with through‐and‐through involvement, and Grade 4 denotes through‐and‐through involvement with clear visibility due to gingival recession.

### 2.3 Data Collection and Variables

Patient‐level and implant‐level data were extracted from electronic health records. Variables included: age at implant placement, sex, smoking status (current/non‐smoker), presence of diabetes mellitus, presence of hypertension, implant arch (maxilla/mandible), and tooth location (anterior/posterior). These variables were recorded as of the date of implant placement.

### 2.4 Outcome Variable

The primary outcome was implant failure, defined as implant removal for any reason (CDT code D6100). The follow‐up time for each implant was calculated from the date of placement to either the date of failure or the date of the last recorded patient visit (censoring). We also conducted a sub‐analysis of failure timing, defined as “early failure” (≤ 1 year) and “late failure” (> 1 year). The treatment outcome was treated as a binary variable (survival/failure) for the analysis of failure rates and as a time‐to‐event variable for the survival analysis.

### 2.5 Statistical Analysis

A multi‐step statistical approach was employed to control for confounding and data clustering.

#### 2.5.1 Propensity Score Matching (PSM)

To address baseline imbalances between the risk and control groups, we performed a 1:4 nearest‐neighbor propensity score matching without replacement. The propensity score was calculated using a logistic regression model based on age, sex, smoking status, diabetes, and implant arch. Standardized mean differences (SMD) were used to assess covariate balance before and after matching, with an SMD < 0.1 indicating adequate balance.

#### 2.5.2 Survival Analysis

On the matched cohort, Kaplan–Meier curves were generated to visualize survival probabilities, and the Log‐Rank test was used to compare survival distributions.

#### 2.5.3 Multilevel Mixed‐Effects Cox Regression

To account for the fact that multiple implants from the same patient are not independent observations, a multivariable, multilevel mixed‐effects Cox proportional hazards model was used as the primary analysis. This model was applied to the matched cohort to estimate adjusted hazard ratios (aHRs) for implant failure. The exposure group (Mobility/Furcation status) was the primary risk indicator, adjusted for the matched covariates (age, sex, smoking, diabetes, hypertension). Patient ID was included as a random effect to account for patient‐level clustering. All analyses were performed with a significance level of *p* < 0.05. All statistical analyses were performed using SAS version 9.4 (SAS Institute Inc., Cary, NC, USA).

This study was conducted and reported in accordance with the Strengthening the Reporting of Observational Studies in Epidemiology (STROBE) guidelines for cohort studies.

## 3 Results

Initially, 50,565 implants were identified (49,557 control; 1008 risk). After 1:4 propensity score matching, a well‐balanced cohort of 3925 implants was created, consisting of 785 implants from the risk groups and 3140 matched control implants. Table 1 shows the baseline characteristics of the groups before and after matching, confirming that all covariates were well‐balanced in the matched cohort (SMD < 0.1). The mean follow‐up period for the matched cohort was 5.8 ± 3.1 years.

**TABLE 1:** Baseline characteristics.

Characteristic	Before matching (risk vs. control)	After matching (risk vs. control)	SMD (after)
N (Implants)	785 vs. 49,557	785 vs. 3140	—
Age (Mean, SD)	65.3 (10.7) vs. 61.2 (12.5)	65.1 (10.6) vs. 65.4 (10.5)	0.03
Female (%)	56.6% vs. 55.0%	56.6% vs. 57.1%	0.01
Current smoker (%)	23.2% vs. 18.0%	23.2% vs. 22.9%	0.01
Diabetes (%)	16.4% vs. 12.0%	16.4% vs. 16.8%	0.01
Hypertension (%)	42.5% vs. 37.0%	42.5% vs. 43.1%	0.01
Maxilla (%)	55.0% vs. 59.0%	55.0% vs. 54.8%	< 0.001

The implant outcomes for the matched cohort are shown in Table 2. The control group had a failure rate of 1.37%, while all risk groups showed significantly higher failure rates, ranging from 2.53% to 4.55%.

**TABLE 2:** Implant outcomes.

Group	Total implants	Failed implants	Failure rate	*p*
Mobility Class 2	498	16	3.21%	< 0.001
Mobility Class 3	110	5	4.55%	
Furcation Grade 3	158	4	2.53%	
Furcation Grade 4	19	1	5.26%	
Matched Control	3140	43	1.37%	

**FIGURE 1:**
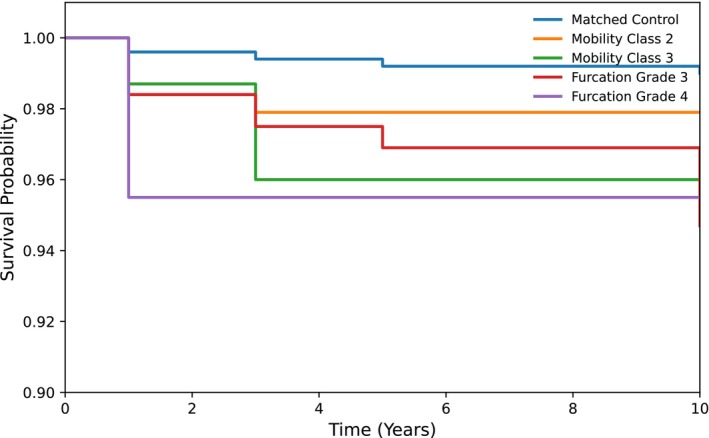
Kaplan–Meier survival curves. This figure displays the Kaplan–Meier survival curves, comparing the 10‐year survival probability of dental implants across the different study groups. The y‐axis represents the estimated probability of implant survival, while the x‐axis represents the follow‐up time in years. The analysis includes implants placed in sites with a pre‐extraction history of severe mobility (Class 2 or 3), advanced furcation involvement (Grade 3 or 4), and a propensity‐score matched control group. The curves visually demonstrate a significantly lower survival probability for all risk groups when compared to the matched control group throughout the 10‐year observation period (Log‐Rank test, *p* < 0.001).

The Kaplan–Meier survival curves (Figure 1) showed significantly lower survival probabilities for all risk groups compared to the matched controls (Log‐Rank test, *p* < 0.001). The estimated 10‐year survival probability was 98.9% for the control group, compared to 94.6% for Mobility Class 2 and 95.5% for Furcation Grade 4 (Table 3).

**TABLE 3:** Estimated survival probability.

Group	1‐year	3‐year	5‐year	10‐year
Matched control	99.6%	99.4%	99.2%	98.9%
Mobility Class 2	98.7%	97.5%	96.9%	94.6%
Mobility Class 3	98.7%	96.0%	96.0%	96.0%
Furcation Grade 3	98.4%	97.9%	97.9%	97.9%
Furcation Grade 4	95.5%	95.5%	95.5%	95.5%

The primary analysis using the multilevel mixed‐effects Cox model (Table 4) confirmed these findings. After adjusting for confounders and patient‐level clustering, all risk groups had a significantly higher hazard of implant failure compared to the matched control group. The risk was highest for Mobility Class 3 (aHR = 3.8) and Furcation Grade 4 (aHR = 4.5).

**TABLE 4:** Multilevel mixed‐effects Cox regression.

Covariate (risk group)	Adjusted hazard ratio (aHR)	95% CI for aHR	*p*
Mobility Class 2	2.3	(1.2–4.4)	< 0.01
Mobility Class 3	3.8	(1.9–7.5)	< 0.01
Furcation Grade 3	3.0	(1.4–6.5)	< 0.01
Furcation Grade 4	4.5	(2.1–9.8)	< 0.01

Abbreviation: aHR, adjusted Hazard Ratio.

The analysis of failure timing revealed that 45% of failures in the risk groups occurred within the first year, compared to 31% in the control group.

## 4 Discussion

This study provides evidence that the pre‐extraction periodontal status of a specific tooth site is significantly associated with future implant failure. Rather than acting as completely independent biological risk factors isolated from the patient's periodontal disease, severe tooth mobility and advanced furcation involvement likely serve as distinct clinical markers for profound localized alveolar bone destruction, compromised bone quality, and a high individual susceptibility to periodontitis. By employing propensity score matching and a multilevel mixed‐effects model, we have addressed the major methodological limitations of previous analyses in this area. Our primary hypothesis was confirmed, as implants placed in sites with a history of severe mobility or advanced furcation involvement demonstrated a significantly higher hazard of failure.

While the use of a multivariable, mixed‐effects model helps adjust for recorded patient‐level factors like smoking or diabetes, it is important to acknowledge that critical unmeasured confounders remain. Specifically, the database did not allow us to consistently classify the overall severity of the patients' generalized periodontitis, nor could we confirm whether active periodontal therapy was successfully completed prior to implant insertion. Therefore, our statistical findings represent a strong association, but cannot definitively prove that local site history is an independent risk factor devoid of the influence of untreated or severe generalized periodontitis. The adjusted hazard ratios, ranging from 2.3 to 4.5, quantify the risk imparted by the local site's history. This adds a critical layer of site‐specific detail to the consensus that a general history of periodontitis elevates implant risk (Annunziata et al. 2025; Carra et al. 2021; Serroni et al. 2024). This study's unique contribution is its focus on the local site, demonstrating that the endpoint of the periodontal disease process—a tooth deemed hopeless due to extreme attachment loss—imparts a distinct and quantifiable risk to the subsequent implant. This is likely explained by the shared etiology of periodontitis and peri‐implantitis, where a severely compromised site may harbor a more virulent residual bacterial load or possess a diminished local healing capacity, predisposing it to future pathology (Smith et al. 2017; Heitz‐Mayfield and Lang 2010). The observed failure rates are likely not caused by the previous tooth's mobility per se, but by the sequelae of that mobility: extensive alveolar bone destruction and poor bone quality. Sites with prior Class 3 mobility or Grade 4 furcation often require extensive grafting. Thus, the pre‐extraction tooth status serves as a critical historical red flag for sites that will present with compromised bone architecture.

A clinically significant “dose–response” relationship was observed, where the hazard of failure generally increased with the severity of the pre‐existing condition. This validates the clinical intuition that replacing a “hopeless” tooth with an implant carries a greater risk. This concept directly extends the principles of tooth‐specific prognosis (McGuire and Nunn 1996) to the prognosis of the implant intended to replace it. These findings have direct implications for treatment planning and patient consent. The pre‐extraction diagnosis should be considered a significant clinical marker warranting a detailed discussion with the patient about the elevated probability of failure. This may influence clinical decisions, such as favoring delayed placement and more rigorous site decontamination protocols.

The findings of this study have significant clinical implications for daily practice, refining risk assessment by incorporating not only a general patient‐level history of periodontitis but also the specific, localized history of the implant site itself. Clinicians can now use this evidence to inform patients more accurately that a prior history of severe mobility or furcation involvement at a tooth site is associated with a 2.3 to 4.5 times greater hazard of future implant failure, even if other systemic factors are well managed. While the absolute difference in survival percentages might appear modest (approximately 3%–4%), in the context of dental implant therapy where baseline success rates are very high, this relative increase represents a substantial deviation from the standard of care. The Hazard Ratio, therefore, serves as the more sensitive and clinically relevant metric for risk assessment. This quantified risk is a crucial component of the informed consent process, ensuring patients have realistic expectations about long‐term prognosis. Consequently, these results should prompt clinicians to adopt more cautious treatment planning for such compromised sites, potentially favoring delayed implant placement over immediate protocols to allow for more thorough site decontamination and soft tissue healing and reinforcing the absolute necessity of enrolling these high‐risk patients in a rigorous, lifelong supportive care program, which has been shown to be critical for mitigating risk in periodontally susceptible populations (Lin et al. 2019; Jepsen et al. 2015).

The primary strength of this study lies in its robust methodological design, specifically implemented to address common sources of bias in retrospective research. By employing propensity score matching, the analysis successfully created well‐balanced risk and control groups, minimizing the confounding effects of key patient‐level variables such as age, smoking, and systemic diseases. Furthermore, the use of a multilevel mixed‐effects Cox regression model is a significant advancement, as it appropriately accounts for the clustered nature of the data where multiple implants are nested within individual patients. This rigorous statistical approach, combined with the large, multicenter cohort that enhances the generalizability of the findings, allows for a more confident conclusion that a history of severe localized periodontal disease is a clinical marker of implant failure.

This study has limitations inherent to its retrospective design. We could not control for variables such as surgical protocols, prosthetic design, or compliance with supportive periodontal therapy (SPT). Data on bone grafting and pre‐operative bone levels were not uniformly available and could represent unmeasured confounders. Furthermore, the outcome of implant removal does not distinguish between biological and mechanical causes. Additionally, the mean follow‐up period of 5.8 years, while sufficient to detect early and mid‐term failures, limits the assessment of late‐stage complications that may arise after a decade of function. Finally, the small sample size in the Furcation Grade 4 group results in a wide confidence interval, and this specific risk estimate should be interpreted with caution. A crucial consideration is that we could not reliably assess the overall periodontitis stage of every patient in the control group. Therefore, our analysis compares severely compromised sites (often in periodontitis‐susceptible patients) to a matched control group that likely has a lower overall burden of periodontal disease. We acknowledge that it is difficult to fully separate patient‐level susceptibility from local site severity in a retrospective design. Also, in a clinical setting, it is often impossible to fully decouple a patient's systemic susceptibility from the severe local destruction it causes. While we matched for a history of periodontitis, the ‘Risk’ group (with Grade 4 furcation/Class 3 mobility) likely represents a phenotype with higher susceptibility to bone loss than the controls. Therefore, the pre‐extraction status of the tooth (e.g., Grade 4 furcation) serves as a vital clinical marker for this susceptibility. Even if the observed risk is partly systemic, identifying these specific ‘hopeless’ teeth helps clinicians flag the patients at the highest risk for future implant failure.

## 5 Conclusions

Within the limitations of this study, this large, propensity‐matched cohort analysis demonstrates that a pre‐extraction history of severe tooth mobility or advanced furcation involvement is significantly associated with subsequent dental implant failure. The hazard of failure was 2.3–4.5 times greater for implants placed in these compromised sites compared to those placed in matched control sites. Rather than acting as purely independent biological causes, these pre‐extraction clinical signs likely serve as critical historical markers for severe localized alveolar bone destruction, compromised local anatomy, and heightened patient susceptibility to periodontitis. These findings underscore the importance of comprehensive site‐specific risk assessment and highlight that the legacy of severe localized periodontal destruction remains a crucial factor in treatment planning, site preparation, and patient counseling prior to implant placement.

## Author Contributions

**Georgios S. Chatzopoulos:** conceptualization, methodology, software, data curation, investigation, writing – original draft, writing – review and editing, validation, visualization, formal analysis, resources, project administration.

## Ethics Statement

The study protocol was reviewed by the Institutional Review Board of the University of Minnesota, which waived the need for ethical review and approval (#STUDY00016865, 10/10/2022).

## Consent

Informed consent was waived due to the retrospective design of the study.

## Conflicts of Interest

The authors declare no conflicts of interest.

## Supporting information


**Data S1:** STROBE Statement—Checklist of items that should be included in reports of cohort studies.

## Data Availability

The data that support the findings of this study are available from the corresponding author upon reasonable request.

## References

[clr70127-bib-0001] Annunziata, M. , G. Cecoro , A. Guida , et al. 2025. “Effectiveness of Implant Therapy in Patients With and Without a History of Periodontitis: A Systematic Review With Meta‐Analysis of Prospective Cohort Studies.” Journal of Periodontal Research 60, no. 2: 524–543. 39466662 10.1111/jre.13351 PMC12312821

[clr70127-bib-0002] Carra, M. C. , H. Rangé , P. J. Swerts , K. Tuand , K. Vandamme , and P. Bouchard . 2021. “Effectiveness of Implant‐Supported Fixed Partial Denture in Patients With History of Periodontitis: A Systematic Review and Meta‐Analysis.” Journal of Clinical Periodontology 48, no. 8: 1083–1104. 10.1111/jcpe.13481 34775625

[clr70127-bib-0003] Giok, K. C. , S. K. Veettil , and R. K. Menon . 2024. “Risk Factors for Peri‐Implantitis: An Umbrella Review of Meta‐Analyses of Observational Studies and Assessment of Biases.” Journal of Dentistry 146: 105065. 38762079 10.1016/j.jdent.2024.105065

[clr70127-bib-0004] Glickman, I. 1972. Clinical Periodontology: Prevention, Diagnosis, and Treatment of Periodontal Disease in the Practice of General Dentistry. 4th ed, 242–245. Saunders.

[clr70127-bib-0005] Heitz‐Mayfield, L. J. , and N. P. Lang . 2010. “Comparative Biology of Chronic and Aggressive Periodontitis vs. Peri‐Implantitis.” Periodontology 2000 53, no. 1: 167–181. 20403112 10.1111/j.1600-0757.2010.00348.x

[clr70127-bib-0006] Jepsen, S. , T. Berglundh , R. Genco , et al. 2015. “Primary Prevention of Peri‐Implantitis: Managing Peri‐Implant Mucositis.” Journal of Clinical Periodontology 42: S152–S157. 25626479 10.1111/jcpe.12369

[clr70127-bib-0007] Karoussis, I. K. , G. E. Salvi , L. J. Heitz‐Mayfield , U. Brägger , C. H. Hämmerle , and N. P. Lang . 2003. “Long‐Term Implant Prognoses in Patients With and Without a History of Chronic Periodontitis: A 10‐Year Prospective Cohort Study of the ITI Dental Implant System.” Clinical Oral Implants Research 14, no. 3: 329–339. 12755783 10.1034/j.1600-0501.000.00934.x

[clr70127-bib-0008] Lin, C. Y. , Z. Chen , W. L. Pan , and H. L. Wang . 2019. “The Effect of Supportive Care in Preventing Peri‐Implant Diseases and Implant Loss: A Systematic Review and Meta‐Analysis.” Clinical Oral Implants Research 30, no. 8: 771–787. 10.1111/clr.13496 31231883

[clr70127-bib-0009] Lin, C. Y. , Z. Chen , W. L. Pan , and H. L. Wang . 2020. “Is History of Periodontal Disease Still a Negative Risk Indicator for Peri‐Implant Health Under Supportive Post‐Implant Treatment Coverage? A Systematic Review and Meta‐Analysis.” International Journal of Oral & Maxillofacial Implants 35, no. 1: 52–62. 31923289 10.11607/jomi.7714

[clr70127-bib-0010] McGuire, M. K. , and M. E. Nunn . 1996. “Prognosis Versus Actual Outcome. II. The Effectiveness of Clinical Parameters in Developing an Accurate Prognosis.” Journal of Periodontology 67, no. 7: 658–665. 8832476 10.1902/jop.1996.67.7.658

[clr70127-bib-0011] Monje, A. , L. Aranda , K. T. Diaz , et al. 2016. “Impact of Maintenance Therapy for the Prevention of Peri‐Implant Diseases: A Systematic Review and Meta‐Analysis.” Journal of Dental Research 95, no. 4: 372–379. 26701350 10.1177/0022034515622432

[clr70127-bib-0012] Pjetursson, B. E. , K. Tan , N. P. Lang , U. Brägger , M. Egger , and M. Zwahlen . 2007. “A Systematic Review of the Survival and Complication Rates of Implant‐Supported Single Crowns (SCs) After an Observation Period of at Least 5 Years.” Clinical Oral Implants Research 18: 63–74.17594371

[clr70127-bib-0013] Renvert, S. , and G. R. Persson . 2009. “Periodontitis as a Potential Risk Factor for Peri‐Implantitis.” Journal of Clinical Periodontology 36: 9–14. 19432626 10.1111/j.1600-051X.2009.01416.x

[clr70127-bib-0014] Roccuzzo, M. , L. Bonino , M. Aglietta , and P. Dalmasso . 2012. “Ten‐Year Results of a Three‐Arm Prospective Cohort Study on Implants in Periodontally Compromised Patients. Part 2: Clinical Results.” Clinical Oral Implants Research 23, no. 4: 389–395. 22092445 10.1111/j.1600-0501.2011.02309.x

[clr70127-bib-0015] Roccuzzo, M. , N. de Angelis , L. Bonino , and M. Aglietta . 2010. “Ten‐Year Results of a Three‐Arm Prospective Cohort Study on Implants in Periodontally Compromised Patients. Part 1: Implant Loss and Radiographic Bone Loss.” Clinical Oral Implants Research 21, no. 5: 490–496. 20337668 10.1111/j.1600-0501.2009.01886.x

[clr70127-bib-0016] Schwarz, F. , J. Derks , A. Monje , and H. L. Wang . 2018. “Peri‐Implantitis.” Journal of Clinical Periodontology 45: S246–S266. 29926484 10.1111/jcpe.12954

[clr70127-bib-0017] Serroni, M. , W. S. Borgnakke , L. Romano , et al. 2024. “History of Periodontitis as a Risk Factor for Implant Failure and Incidence of Peri‐Implantitis: A Systematic Review, Meta‐Analysis, and Trial Sequential Analysis of Prospective Cohort Studies.” Clinical Implant Dentistry and Related Research 26: 1–17. 10.1111/cid.13330 38720611

[clr70127-bib-0018] Smith, M. M. , E. T. Knight , L. Al‐Harthi , and J. W. Leichter . 2017. “Chronic Periodontitis and Implant Dentistry.” Periodontology 2000 74, no. 1: 63–73. 28429486 10.1111/prd.12190

[clr70127-bib-0019] Tomasi, C. , J. P. Albouy , D. Schaller , R. C. Navarro , and J. Derks . 2021. “Efficacy of Rehabilitation of Stage IV Periodontitis Patients With Full‐Arch Fixed Prostheses: Tooth‐Supported Versus Implant‐Supported—A Systematic Review.” Journal of Clinical Periodontology 48, no. 9: 1249–1267. 10.1111/jcpe.13511 34761430

