# Supplementary material for: Prior Tooth Mobility and Furcation Involvement Are Associated With Higher Dental Implant Failure Rates: A Propensity‐Matched Cohort Study
Source: Clin Oral Implants Res. 2026 Apr 3;37(7):835–40. doi: 10.1111/clr.70127 (PMC13340521; doi:10.1111/clr.70127)
Supplement: Supplementary file 1 — Data S1: STROBE Statement—Checklist of items that should be included in reports of cohort studies. [file CLR-37-835-s001.docx]

**STROBE Statement—Checklist of items that should be included in reports of cohort studies**

| **Item No** | **Recommendation** | **Location in Manuscript** |
| --- | --- | --- |
| **Title and abstract** |  |  |
| 1 | (a) Indicate the study’s design with a commonly used term in the title or the abstract. | **Title & Abstract:** The term "Propensity-Matched Cohort Study" is used in the title and "retrospective cohort study" is used in the abstract. |
|  | (b) Provide in the abstract an informative and balanced summary of what was done and what was found. | **Abstract:** The abstract provides a structured summary covering Objectives, Material and Methods, Results, and Conclusions. |
| **Introduction** |  |  |
| 2 | **Background/rationale:** Explain the scientific background and rationale for the investigation being reported. | **Introduction, Para 1-4:** The background on implant success, risk factors like periodontitis, and the specific knowledge gap regarding site-specific predictors is clearly explained. |
| 3 | **Objectives:** State specific objectives, including any prespecified hypotheses. | **Introduction, Para 5:** The primary aim and the specific hypothesis are stated in the final paragraph. |
| **Methods** |  |  |
| 4 | **Study design:** Present key elements of study design early in the paper. | **Section 2.1 (Study Design and Population):** The design is stated as a "retrospective cohort analysis." |
| 5 | **Setting:** Describe the setting, locations, and relevant dates, including periods of recruitment, exposure, follow-up, and data collection. | **Section 2.1:** The study setting (BigMouth network universities) and the data collection period (2011 to 2022) are described. |
| 6 | **Participants:** (a) Give the eligibility criteria, and the sources and methods of selection of participants. | **Section 2.1:** Eligibility criteria (adult patients with at least one endosteal implant) and selection methods (using CDT codes from electronic records) are detailed. |
| 7 | **Variables:** Clearly define all outcomes, exposures, predictors, potential confounders, and effect modifiers. Give diagnostic criteria, if applicable. | **Sections 2.2, 2.3, 2.4:** Exposures (Mobility/Furcation groups), Outcome (implant failure), and other variables (demographics, health conditions) are clearly defined. |
| 8 | **Data sources/ measurement:** For each variable of interest, give sources of data and details of methods of assessment (measurement). | **Sections 2.1-2.4:** The sources (electronic health records, periodontal charting) and methods of identification (CDT codes) are specified for all key variables. |
| 9 | **Bias:** Describe any efforts to address potential sources of bias. | **Section 2.5 (Statistical Analysis):** Efforts to control for confounding bias (Propensity Score Matching) and clustering bias (multilevel mixed-effects model) are described. |
| 10 | **Study size:** Explain how the study size was arrived at. | **Not Reported:** As is common for retrospective studies, the sample size was based on the available data within the specified timeframe. No a priori sample size calculation was mentioned. |
| 11 | **Quantitative variables:** Explain how quantitative variables were handled in the analyses. If applicable, describe which groupings were chosen and why. | **Section 2.5:** The handling of quantitative variables (e.g., age) as continuous inputs for the propensity score model is implicitly described. |
| 12 | **Statistical methods:** (a) Describe all statistical methods, including those used to control for confounding. | **Section 2.5:** A detailed description of the statistical approach is provided, including propensity score matching, Kaplan-Meier analysis, Log-Rank test, and the multilevel mixed-effects Cox model. |
|  | (b) Describe any methods used to examine subgroups and interactions. | **Section 2.4:** The sub-analysis of failure timing ("early" vs. "late") is mentioned. |
|  | (c) Explain how missing data were addressed. | **Not Reported:** The manuscript does not specify the method for handling missing data. |
|  | (d) If applicable, explain how loss to follow-up was addressed. | **Section 2.4:** Loss to follow-up is handled by censoring observations at the "date of the last recorded patient visit." |
|  | (e) Describe any sensitivity analyses. | **Not Reported:** No sensitivity analyses were mentioned. |
| **Results** |  |  |
| 13 | **Participants:** (a) Report numbers of individuals at each stage of study—eg, numbers potentially eligible, examined for eligibility, confirmed eligible, included in the study, completing follow-up, and analysed. | **Results, Para 1:** The flow of participants is described numerically from the initial identification (50,565 implants) to the final matched cohort (3,925 implants). A flow diagram is not included but the numbers are clear. |
|  | (b) Give reasons for non-participation at each stage. | **Not Applicable:** This is a retrospective database study, so non-participation is not relevant in the traditional sense. |
| 14 | **Descriptive data:** (a) Report characteristics of study participants. | **Results, Para 1 & Table 1:** Baseline demographic and clinical characteristics are presented for the cohorts before and after matching. |
|  | (b) Indicate number of participants with missing data for each variable of interest. | **Not Reported.** |
|  | (c) Summarise follow-up time (eg, average and total amount). | **Results, Para 1:** The mean follow-up period (5.8 ± 3.1 years) for the matched cohort is reported. |
| 15 | **Outcome data:** Report numbers of outcome events or summary measures over time. | **Results, Para 2 & Table 2:** The number of failed implants and failure rates are reported for each group. **Table 3** provides survival probabilities over time. |
| 16 | **Main results:** (a) Give unadjusted estimates and, if applicable, confounder-adjusted estimates and their precision (eg, 95% confidence interval). Make clear which confounders were adjusted for and why. | **Results, Para 4 & Table 4:** Confounder-adjusted hazard ratios (aHR) with 95% confidence intervals are presented as the main outcome. The adjusted variables are implicitly those used in the PSM. |
|  | (b) Report category boundaries when continuous variables were categorised. | **Not Applicable:** The main continuous variable (age) was not categorized for the primary analysis. |
|  | (c) If relevant, consider translating estimates of relative risk into absolute risk for a meaningful time period. | **Table 3:** Absolute risk is presented as survival probabilities at 1, 3, 5, and 10 years. |
| 17 | **Other analyses:** Report other analyses done—eg analyses of subgroups and interactions, and sensitivity analyses. | **Results, Para 5:** The sub-analysis on the timing of implant failure (early vs. late) is reported. |
| **Discussion** |  |  |
| 18 | **Key results:** Summarise key results with reference to study objectives. | **Discussion, Para 1:** The discussion begins with a clear summary of the main findings in relation to the study's primary hypothesis. |
| 19 | **Limitations:** Discuss limitations of the study, taking into account sources of potential bias or imprecision. Discuss both direction and magnitude of any potential bias. | **Discussion, "Limitations" section:** A dedicated paragraph details several limitations, including the retrospective design, unmeasured confounders (grafting, SPT compliance), and small sample size in one subgroup. |
| 20 | **Interpretation:** Give a cautious overall interpretation of results considering objectives, limitations, multiplicity of analyses, results from similar studies, and other relevant evidence. | **Discussion Section:** The findings are interpreted cautiously and contextualized with existing literature and potential biological mechanisms. |
| 21 | **Generalisability:** Discuss the generalisability (external validity) of the study results. | **Discussion, "Strengths" section:** Generalizability is addressed by noting the "large, multicenter cohort." |
| **Other information** |  |  |
| 22 | **Funding:** Give the source of funding and the role of the funders. | **Front Matter:** A funding statement is provided, indicating "None." |
